# The effects of postprandial glucose and insulin levels on postprandial endothelial function in subjects with normal glucose tolerance

**DOI:** 10.1186/1475-2840-11-98

**Published:** 2012-08-14

**Authors:** Kazunari Suzuki, Kentaro Watanabe, Shoko Futami-Suda, Hiroyuki Yano, Masayuki Motoyama, Noriaki Matsumura, Yoshimasa Igari, Tatsuya Suzuki, Hiroshi Nakano, Kenzo Oba

**Affiliations:** 1 Department of Internal Medicine (Divisions of Cardiology, Hepatology, Geriatrics, and Integrated Medicine), Nippon Medical School, Tokyo, Japan

**Keywords:** Flow-mediated dilation, Carbohydrate content, Postprandial, Glucose metabolism, Non-diabetic individuals

## Abstract

**Background:**

Previous studies have demonstrated that postprandial hyperglycemia attenuates brachial artery flow-mediated dilation (FMD) in prediabetic patients, in diabetic patients, and even in normal subjects. We have previously reported that postprandial hyperinsulinemia also attenuates FMD. In the present study we evaluated the relationship between different degrees of postprandial attenuation of FMD induced by postprandial hyperglycemia and hyperinsulinemia and differences in ingested carbohydrate content in non-diabetic individuals.

**Methods:**

Thirty-seven healthy subjects with no family history of diabetes were divided into 3 groups: a 75-g oral glucose loading group (OG group) (n = 14), a test meal group (TM group) (n = 12; 400 kcal, carbohydrate content 40.7 g), and a control group (n = 11). The FMD was measured at preload (FMD0) and at 60 minutes (FMD60) and 120 (FMD120) minutes after loading. Plasma glucose (PG) and immunoreactive insulin (IRI) levels were determined at preload (PG0, IRI0) and at 30 (PG30, IRI30), 60 (PG60, IRI60), and 120 (PG120, IRI120) minutes after loading.

**Result:**

Percentage decreases from FMD0 to FMD60 were significantly greater in the TM group (−21.19% ± 17.90%; P < 0.001) and the OG group (−17.59% ± 26.64%) than in the control group (6.46% ± 9.17%; P < 0.01), whereas no significant difference was observed between the TM and OG groups. In contrast, the percentage decrease from FMD0 to FMD120 was significantly greater in the OG group (−18.91% ± 16.58%) than in the control group (6.78% ± 11.43%; P < 0.001) or the TM group (5.22% ± 37.22%; P < 0.05), but no significant difference was observed between the control and TM groups. The FMD60 was significantly correlated with HOMA-IR (r = −0.389; P < 0.05). In contrast, FMD120 was significantly correlated with IRI60 (r = −0.462; P < 0.05) and the AUC of IRI (r = −0.468; P < 0.05). Furthermore, the percentage change from FMD0 to FMD120 was significantly correlated with the CV of PG (r = 0.404; P < 0.05), IRI60 (r = 0.401; p < 0.05) and the AUC of IRI (r = 0.427; P < 0.05). No significant correlation was observed between any other FMDs and glucose metabolic variables.

**Conclusion:**

Differences in the attenuation of postprandial FMD induced by different postprandial insulin levels may occur a long time postprandially but not shortly after a meal.

## Introduction

Postprandial hyperglycemia is a major risk factor for morbidity and mortality from cardiovascular disease in diabetes [1,2]. Furthermore, in prediabetes, the postprandial glucose level, but not the impaired fasting glucose tolerance, is an independent risk factor for cardiovascular disease [3]. Postprandial hyperglycemia is independently related to the risk of cardiovascular disease in patients with prediabetes or diabetes. Furthermore, in these patients postprandial hyperglycemia is more strongly related to the progression of atherosclerosis than is fasting hyperglycemia [4]. Vascular endothelial dysfunction plays an important early role in atherosclerosis [5] and can be assessed by measuring flow-mediated dilation (FMD) of the brachial artery with ultrasonography during reactive hyperemia [6,7]. Attenuation of brachial artery FMD is induced by postprandial hyperglycemia in subjects with normal glucose tolerance and in prediabetic or diabetic patients, and FMD is negatively correlated with plasma glucose (PG) levels after oral glucose loading [8-11]. We have previously reported that postprandial hyperinsulinemia also induces attenuation of FMD in persons with normal glucose tolerance [12]. Several studies have evaluated differences in FMD attenuation after meals with different carbohydrate contents [13-17]. Although these studies have found that the attenuation of postprandial FMD is affected by postprandial lipid metabolism [14-17], how it is affected by glucose metabolism after meals with different carbohydrate contents remains unclear.

Therefore, in the present study, we evaluated differences in the attenuation of postprandial FMD induced by postprandial hyperglycemia and hyperinsulinemia after the intake of meals with different carbohydrate contents in non-diabtetic subjects.

## Methods

### Study subjects

Thirty-seven healthy Japanese volunteers aged 22 to 61 years (19 men and 18 women; mean age, 31.8 ± 9.2 years) with no family history of diabetes were recruited. Before enrollment, written informed consent was obtained from all subjects. All subjects underwent medical checkups and denied a history of dyslipidemia, diabetes mellitus, hypertension, or other chronic diseases. Normal glucose tolerance was defined as a fasting PG level of <6.1 mmol/L and a 2-hour value of <7.8 mmol/L on the 75-g oral glucose loading test, on the basis of the Report of the Committee on the Classification and Diagnostic Criteria of Diabetes Mellitus of the Japanese Diabetes Society [18]. Normal blood pressure was defined as a blood pressure of <140/90 mmHg that was measured in a hospital, on the basis of the Japanese Society of Hypertension Committee for Guidelines for the Management of Hypertension [19]. Normal lipid metabolism was defined as low-density lipoprotein cholesterol (LDL-C) level of <140 mg/dL (3.63 mmol/L), a high-density lipoprotein cholesterol (HDL-C) level of ≥ 40 mg/dL (1.04 mmol/L), and a triglyceride (TG) level <150 mg/dL (1.70 mmol/L), on the basis of the Executive Summary of Japan Atherosclerosis Society Guideline for Diagnosis and Prevention of Atherosclerotic Cardiovascular Disease of Japanese [20].

### Study design

The study was designed in compliance with the ethics regulations set out by the Helsinki Declaration. Reporting of the study conforms to the STROBE (Strengthening the Reporting of Observational Studies in Epidemiology) guidelines, along with references to STROBE and the broader EQUATOR (Enhancing the Quality and Transparency of Health Research) guidelines.

The 37 study subjects were divided into 3 groups on the basis of carbohydrate intake: (1) a test meal group (TM group) (intake = 40.7 g of carbohydrates, 400 kcal; n = 12), (2) a 75-g oral glucose load group (OG group) (intake = 75 g of carbohydrates, 300 kcal; n = 14), and (3) an overnight fasting group (control group) (n = 11) (Table1). The subjects of the OG and control groups had previously been included in our study of postprandial attenuation of FMD [12]. The test meal for the TM group (Table2) was a commercial energy-supplement food (CalorieMate®, Otsuka Pharmaceutical Co., Ltd., Tokyo, Japan). The subjects’ baseline clinical characteristics and biochemical variables, including glucose metabolic variables, were evaluated at 9 a.m. after at least 12 hours of overnight fasting. The glucose metabolic variables selected were PG levels and plasma immunoreactive insulin (IRI) levels. The PG and IRI levels of all groups after overnight fasting were defined as PG0 and IRI0, respectively. After the biochemical variables had been evaluated after overnight fasting, the subjects of the OG group ingested 75 g of glucose, and the TM group ingested the test meal with 500 ml of water within 10 minutes. The PG and IRI levels in the OG and TM groups were then evaluated at 30 (PG30 and IRI30), 60 (PG60 and IRI60), and 120 minutes (PG120 and IRI120) after loading. The area under the curve (AUC) and the coefficient of variation (CV) of the PG or IRI level were determined from 4 measurement points. The FMD of the right brachial artery was evaluated by means of A- and B-mode ultrasonography with a 10-MHz linear-array transducer (UNEXEF18G, UNEX Corp., Nagoya, Japan). This transducer can accurately capture the target artery from 3 directions and can continuously measure the FMD of the same position with B-mode ultrasound images. For the hyperemia scan, the conduit artery was measured continuously for 180 seconds after cuff deflation. For FMD measurements with the transducer, the interobserver correlation coefficient was r = 0.961 (P < 0.001), and the intraobserver variability was 2.4%. The subjects were instructed to abstain from smoking and caffeine consumption for at least 8 hours before the start of the study and to lie down for 15 minutes. The FMD was measured according to a report of the International Brachial Artery Reactivity Task Force [6]. The baseline diameter of the brachial artery was defined as its mean diameter 5 cm proximal to the elbow joint during 4 consecutive diastoles on an electrocardiogram before hyperemia. After this baseline diameter had been determined, forearm hyperemia was produced with sphygmomanometric cuff inflation to 50 mmHg greater than the systolic blood pressure for 5 minutes. After the cuff had been deflated, the maximum diameter of the brachial artery after hyperemia was measured. The rate of change in diameter (%) determined from the maximum diameters at baseline and after hyperemia was defined as the FMD. The FMDs of the OG and TM groups determined at preload were designated FMD0s, and those measured 60 and 120 minutes after loading were designated FMD60s and FMD120s, respectively. In the control group FMDs were measured 3 times at 1-hour intervals after the overnight fast and designated FMD0, FMD60, and FMD120. 

**Table 1:** Clinical characteristics of subjects of the 3 groups

**Clinical characteristic**	**Control group (n = 11)**	**TM group (n = 12)**	**OG group (n = 14)**
Sex (men)	7 (63.6)	6 (50.0)	6 (42.9)
Age (years)	33.4 ± 8.4	28.5 ± 5.1	33.4 ± 11.9
BMI (kg/m^2^)	21.94 ± 1.88	22.53 ± 2.46	20.71 ± 2.28
Smoking habits			
None	6 (54.5)	10 (83.3)	5 (35.7)
Recent	2 (18.2)	2 (16.7)	6 (42.9)
Current	3 (27.3)	0 (0)	3 (21.4)
Family history of diabetes	0 (0)	0 (0)	0 (0)
Blood pressure (mm Hg)			
Systolic	104.8 ± 5.2	115.9 ± 11.5^*^	105.5 ± 8.5
Diastolic	61.5 ± 7.0	71.2 ± 9.1^*^	64.3 ± 6.4
TC (mmol/L)	4.55 ± 0.58	4.46 ± 0.61	4.95 ± 0.61
HDL-C (mmol/L)	1.46 ± 0.26	1.63 ± 0.37	1.73 ± 0.42
LDL-C (mmol/L)	2.62 ± 0.50	2.39 ± 0.36	2.74 ± 0.54
TG (mmol/L)	0.99 ± 0.34	0.66 ± 0.29^#^	0.82 ± 0.35
Uric acid (μmol/L)	294.4 ± 36.7	323.2 ± 109.4	315.9 ± 100.6
Serum creatinine (μmol/L)	62.12 ± 13.07	61.00 ± 8.22	65.54 ± 14.11
Fasting PG (mmol/L)	5.09 ± 0.54	4.75 ± 0.62	4.79 ± 0.58
IRI (pmol/L)	38.70 ± 16.35	30.67 ± 13.63	32.71 ± 16.94
HOMA-IR	1.18 ± 0.75	0.92 ± 0.52	1.07 ± 0.73
HbA_1C_ (%)	5.40 ± 0.21	5.21 ± 0.29	5.37 ± 0.26

mean ± SD, n (%).

TM: test meal, OG: 75-g oral glucose loading.

^*^P < 0.05 for TM group vs control and OG groups, ^#^P < 0.05 for TM group vs control group.

FPG: fasting plasma glucose. IRI: immunoreactive insulin. HOMA-IR: homeostasis model assessment of insulin resistance.

**Table 2:** Meal composition, glucose metabolism variables, and FMD of the three groups

**Variables**	**Control group (N = 11)**	**TM group (N = 12)**	**OG group (N = 14)**
(1) Meal composition			
Carbohydrate content (g)	0	40.7	75
Lipid content (g)	0	22.2	0
Total calorie (kcal)	0	400	300
(2) Glucose metabolism variables			
Plasma glucose (mmol/L)			
0 min.	5.16 ± 0.42	4.71 ± 0.37*	4.65 ± 0.43*
30 min.	n/a	5.41 ± 0.97	6.83 ± 1.84^#^
60 min.	n/a	5.16 ± 1.07	6.07 ± 1.61
120 min.	n/a	5.28 ± 0.39	5.05 ± 1.13
AUC of PG [(mmol/L)·hr]	n/a	10.39 ± 1.32	11.66 ± 2.21
CV of PG	n/a	0.12 ± 0.06	0.25 ± 0.10^**##**^
Plasma IRI (pmol/L)			
0 min.	n/a	34.16 ± 18.6	29.67 ± 11.95
30 min.	n/a	204.25 ± 61.65	278.08 ± 109.28^**#**^
60 min.	n/a	144.86 ± 82.76	281.11 ± 155.48^**##**^
120 min.	n/a	76.00 ± 38.32	138.53 ± 62.33^**#**^
AUC of IRI [(pmol/L)·hr]	n/a	257.31 ± 87.04	426.55 ± 126.73^**##**^
CV of IRI	n/a	0.73 ± 0.17	0.88 ± 0.22
(3) FMD (%)			
FMD0	9.93 ± 3.97	11.98 ± 5.53	9.16 ± 1.85
FMD60	10.43 ± 3.71	9.10 ± 3.97	7.53 ± 2.98*
FMD120	10.46 ± 3.73	11.83 ± 5.04	7.46 ± 2.28^#*^

mean ± SD.

^*^p < 0.05 vs. Control group, ^**#**^p < 0.05, ^**##**^p < 0.01 vs TM group.

PG: plasma glucose. IRI: immunoreactive insulin. AUC: area under the curve.

CV: coefficient of variation. FMD: flow-mediated dilation. FMD0, FMD60,

and FMD 120: FMD at pre-load, 60 minutes, and 120 minutes after meal loading.

n/a: not available.

To evaluate differences in how FMD changed among the 3 groups, the percentage change from FMD0 to FMD 60 was calculated as 100 × (FMD60-FMD0)/FMD0, and the change from FMD0 to FMD120 was calculated as 100 × (FMD120-FMD0)/FMD0. To evaluate the correlations between changes in FMD and glucose metabolic variables, the percentage changes in FMD in the 3 groups were calculated as follows: ΔF1% = 100 × (FMD0-FMD60)/FMD0, and ΔF2% = 100 × (FMD0-FMD120)/FMD0.

### Clinical characteristics

After overnight fasting, the clinical characteristics of the subjects were evaluated. These characteristics included sex, age, body-mass index (BMI), smoking habit, family history of diabetes, systolic and diastolic blood pressures, and biochemical variables, including serum total cholesterol (TC), LDL-C, HDL-C, TG, PG, IRI, and HbA_1C_. Serum TC, LDL-C, HDL-C, and TG were measured with an automated analyzer. The PG level was measured with the glucose oxidase method. The plasma IRI level was measured with a radioimmunoassay, and HbA_1C_ (Japan Diabetes Society: JDS) was measured with high-performance liquid chromatography (JDS Lot 3 calibration). The HbA_1c_ (JDS) was converted into HbA1c (NGSP), so that HbA_1c_ (JDS) + 0.4 became equal to HbA1c (NGSP) (%) [21].

### Statistical analysis

The Mann–Whitney *U*-test was used to compare differences in clinical characteristics and FMD among the 3 groups. The Wilcoxon test was used to compare differences in the mean values of FMD0, FMD60, and FMD120. Pearson’s correlation coefficients were used to evaluate correlations between FMD and glucose metabolic variables. Data are presented as means ± SD. Statistical significance was indicated by P < 0.05. All analyses were performed with SPSS for Windows Ver. 12.0 J (SPSS Japan Inc).

## Results

The TG levels in the TM group were significantly lower than those in the control group, but no significant differences in other clinical characteristics were observed among the 3 groups (Table1).

**Figure 1:**
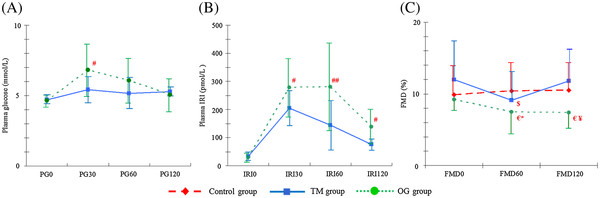
**Changes in PG (A), IRI (B), and FMD (C) in the TM, OG, and control groups.** #P < 0.05, ## P < 0.01 vs. PG or IRI in the TM group, €P < 0.05 vs. FMD0 in the OG group, $P < 0.01 vs. FMD0 and FMD120 in the TM group,*P < 0.05 vs. FMD60 of the control group, \P < 0.05 vs. FMD120 in the TM and control groups.

The PG level in the OG group (Table2, Figure1A) increased markedly during the first 30 minutes after loading but had returned to baseline by 120 minutes. In the TM group, however, PG increased slightly in the first 30 minutes after meal ingestion, then decreased slightly, and finally increased slightly again between 60 and 120 minutes. The PG30 was significantly higher in the OG group than in the TM group, and no significant correlation was observed at any other time. The IRI in the TM group (Table2, Figure1B) increased markedly in the first 30 minutes after meal ingestion and then gradually decreased. In the OG group the IRI showed a similar pattern, but the peak was somewhat delayed. The IRI30, IRI60, and IRI120 were all significantly higher in the OG group than in the TM group. The CV of PG and the AUC of IRI were significantly higher in the OG group than in the TM group. However, neither the AUC of PG nor the CV of the IRI differed significantly between the OG and TM groups (Table2).

The FMDs in the control group did not differ significantly from each other (Table2, Figure1C). In the TM group, however, FMD60 was significantly lower than FMD0 (P < 0.01), but FMD120 did not differ significantly from FMD0. In the OG group, both FMD60 and FMD 120 were significantly lower than FMD0 (both P < 0.05).

**Figure 2:**
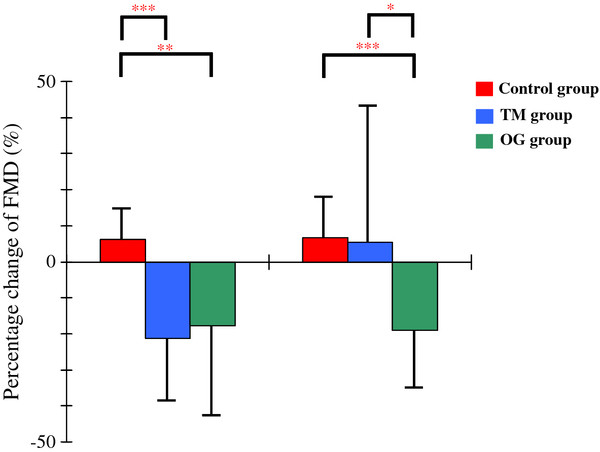
Percentage changes in FMD in the control, TM, and OG groups. From FMD0 to FMD 60: 100 × (FMD60-FMD0)/FMD0. From FMD0 to FMD120: 100 × (FMD120-FMD0)/FMD0. TM group: test meal group, OG group: 75-g oral glucose loading group.

The percentage decrease from FMD0 to FMD60 in the TM group (−21.19% ± 17.90%, P < 0.001) and in the OG (−17.59% ± 26.64%, P < 0.01) group was significantly greater than that in the control group (6.46 ± 9.17%) but did not differ significantly between the TM group and the OG group (Figure2). In contrast, the percentage decrease from FMD0 to FMD120 was significantly greater in the OG group (−18.91% ± 16.58%) than in the control group (6.78% ± 11.43%, P < 0.001) or in the TM group (5.22% ± 37.22%, P < 0.05) but did not differ significantly between the control group and the TM group.

An analysis of FMD0 and glucose metabolic variables showed no significant correlation in either the TM group or the OG group (Table3). However, significant correlations were found of FMD60 with the homeostasis model assessment of insulin resistance (HOMA-IR) and of FMD120 with IRI60 and with the AUC of IRI. No significant correlation was observed between ΔF1% and glucose metabolic variables, but ΔF2% was significantly correlated with the CV of PG (r = 0.404; P = 0.041), IRI60 (r = 0.401; P = 0.042), and the AUC of IRI (r = 0.427; P = 0.03).

**Table 3:** Correlations between FMD and glucose metabolism variables of TM and OG groups

**Glucose metabolism variables**	**FMD0**	**FMD60**	**FMD120**	**ΔF1%**	**ΔF2%**
PG (mmol/L)					
PG30	−0.227	−0.115	−0.281	−0.101	0.279
PG60	−0.166	0.072	−0.221	−0.251	0.295
PG120	0.112	0.115	0.154	−0.032	−0.060
AUC of PG	−0.182	0.002	−0.222	−0.194	0.264
CV of PG	−0.141	−0.170	−0.323	0.047	0.404^*^
IRI (pmol/L)					
IRI30	−0.107	−0.161	−0.238	0.021	0.197
IRI60	−0.260	−0.078	−0.438^*^	−0.250	0.401^*^
IRI120	−0.033	0.217	−0.145	−0.306	0.236
AUC of IRI [(mmol/L)·hr]	−0.253	−0.079	−0.460^*^	−0.247	0.427^*^
CV of IRI [(pmol/L)·hr]	−0.220	−0.306	−0.315	0.035	0.147
HOMA-IR	−0.297	−0.389*	−0.234	0.189	−0.139

^*^P < 0.05.

PG30, PG60, and PG120: plasma glucose at 30, 60, and 120 minutes after meal loading.

IRI30, IRI60, and IRI120: immunoreactive insulin 30, 60, and 120 minutes after meal loading.

ΔF1%: 100 × (FMD0-FMD60)/FMD0. ΔF2%: 100 × (FMD0-FMD120)/FMD0.

HOMA-IR: homeostasis model assessment of insulin resistance.

## Discussion

The present study has demonstrated attenuation of postprandial FMD, both after ingestion of a test meal and after oral glucose loading, which was correlated with variables of glucose metabolism. However, the relationship between the postprandial FMD attenuation and glucose metabolic variables differed between the ingestion of a test meal and oral glucose loading.

Although PG and IRI levels in the OG group were higher than those in the TM group, the FMD60 was similar in both groups. The FMD60 was correlated only with the HOMA-IR, and no significant association was found between HOMA-IR and FMD120. In fact, the change from FMD0 to FMD60 did not differ significantly between the TM group and the OG group. These results suggest that insulin resistance is associated with short-term, but not long-term, attenuation of vascular endothelial function both after test meal ingestion and after oral glucose loading. Oral antidiabetic agents, such as pioglitazone, that decrease insulin resistance also improve endothelial function in patients with diabetes [22] or impaired glucose tolerance [23]. Improvement of fasting FMD is correlated with improvement of insulin resistance [24]. The brachial artery FMD is correlated with the euglycemic hyperinsulinemic glucose clamp-derived glucose metabolic clearance rate in normotensive first-degree relatives of patients with type 2 diabetes [25]. We speculate that the delay in glucose metabolic clearance in patients with insulin resistance induces greater attenuation of postprandial FMD in subjects with insulin resistance than in subjects with normal insulin sensitivity.

In contrast, although FMD showed a continuous decrease in the OG group, it did not decrease in the TM group during study period. The change in FMD from 0 to 120 minutes after loading was significantly correlated with the CV of PG, IRI60, and the AUC of IRI. These findings suggest that FMD120 in our subjects was associated with the magnitude of elevation and fluctuation of both postprandial PG and IRI. Endothelial dysfunction in diabetes has been attributed to elevated oxidative stress levels induced by several mechanisms, especially abnormalities in glucose metabolism [26]. In fact, endothelial dysfunction is improved by oral antidiabetic agents in patients with type 2 diabetes [22,27-29]. These agents ameliorate abnormalities in glucose metabolism and, as a result, reduce oxidative stress in patients with diabetes. Both FMD and PG levels are significantly correlated with plasma levels of thiobarbituric acid reactive substances during oral glucose tolerance testing in subjects with normal glucose tolerance who have first-degree relatives with type 2 diabetes [9] and in subjects with impaired glucose tolerance [10]. Acarbose, nateglinide, and mitiglinide have been reported to decrease postprandial hyperglycemia and to improve endothelial function in patients with type 2 diabetes [27,30,31] and in patients with impaired glucose tolerance [32]. Furthermore, repetitive fluctuations in PG or insulin reportedly enhance monocyte adhesion to the endothelium of the rat thoracic aorta, and stable hyperglycemia or hyperinsulinemia has been found to cause less monocyte adhesion than does repetitive PG fluctuation [33]. These previous findings and the results of our present study suggest that the difference in oxidative stress induced by the difference in postprandial hyperglycemia between the TM and OG groups caused the difference in attenuation of FMD120 in our subjects with normal glucose tolerance.

Our study has also demonstrated that the attenuation of postprandial FMD is significantly associated with plasma insulin levels in subjects with normal glucose tolerance. Plasma insulin is generally considered to have a beneficial effect on vascular endothelial function [34]. In contrast, insulin resistance reduces the bioavailability of endothelial nitric oxide. Insulin resistance decreases the number of endothelial progenitor cells and hinders the vascular repair induced by disturbed PI3K/AKt signaling, reactive oxygen species, inflammation, and adipokines [35]. The continuous infusion of insulin with an euglycemic or hyperglycemic clamp causes attenuation of femoral and brachial artery endothelium-dependent vasodilation 4 hours after baseline, and attenuation of FMD induced by insulin is inhibited by the addition of an antioxidant agent (vitamin C) [36]; however, endothelium-independent vasodilation is not attenuated by insulin. These findings suggest that a high plasma level of insulin in the postprandial state attenuates postprandial FMD and delays the recovery of FMD after absorption of PG.

Our study had several limitations. First, we could not evaluate the effect of postprandial lipid metabolism on postprandial FMD. We had concluded that the effect of 22 g of fat in the meal ingested by the TM group could be ignored. Accordingly, we measured FMD from baseline to 120 minutes after loading following the method of Lavi et al. [13]. However, several studies have shown that postprandial attenuation of FMD induced by fat lasts for more than 120 minutes after fat loading [15,16]. Therefore, a study measuring postprandial FMD for more than 120 minutes after loading is needed to clarify the effects of dietary fat intake observed in the present study. Second, Major-Pedersen et al. have reported, in contrast with our present results, that oral glucose does not induce endothelial dysfunction in healthy persons with mean insulin and PG values of 5.6 mmol/L and 27.2 mmol/L, respectively, 2 hours after a glucose load [37]. However, the study of Major-Petersen et al. and the present study differ in the methods of FMD measurement and the characteristics of subjects. For example, the effects of reactive hyperemia on the sympathetic nerve might differ because Major-Petersen et al. used a sphygmomanometric cuff pressure that was higher (300 mm Hg) than the pressure we used (systolic blood pressure + 50 mm Hg). Also, the subjects of Major-Petersen et al. might have been healthier than ours because they had a lower mean fasting PG level. Furthermore, the peak PG and IRI levels that induced postprandial attenuation of FMD might have appeared less than 30 minutes after loading because the mean age of subjects in our TM group was 28.5 years [38]. Therefore, the conflicting results between the present study and the study of Major-Petersen et al. might be attributed to these differences. Third, we could not assess the relationship between oxidative stress and FMD in our subjects. It is necessary to evaluate the effects of postprandial lipid metabolism, heart rate, blood pressure, and oxidative stress on postprandial FMD. Fourth, the study population of our study was a composite group with a wide range of PG and IRI levels, whereas our study subjects had been identified with medical checkups to have normal glucose tolerance. A cross-over study should be performed to resolve these problems. Finally, because the number of subjects in the present study was small, our results should be clarified with a larger number of subjects.

In summary, our study suggests that attenuation of brachial artery FMD in the postprandial state is correlated with postprandial glucose fluctuation, insulin resistance, and the postprandial serum insulin level in non-diabetic individuals. Furthermore, differences in the attenuation of postprandial vascular endothelial function induced by different postprandial insulin levels may occur a long time postprandially but not shortly after a meal.

Further longitudinal studies are necessary in subjects with normal glucose tolerance to investigate the long-term effects of postprandial glucose metabolism on endothelial function and the possible contribution of this endothelial dysfunction to the development of atherosclerosis.

## Abbreviations

FMD: Flow-mediated Dilation; OG group: The 75-g oral glucose loading group; TM group: Test Meal group; IRI: Immunoreactive Insulin; HOMA-IRhe: Homeostasis Model Assessment of Insulin Resistance; CV: The coefficient of Variation; PG: Plasma Glucose; AUC: The area Under the Curve; TC: Total Cholesterol; LDL-C Low-Density: Lipoprotein Cholesterol; HDL-C: High-Density Lipoprotein Cholesterol; TG: Triglyceride; BMI: Body-Mass Index; JDS: Japan Diabetes Society.

## Competing interests

The authors declare that they have no competing interests.

## Authors’ contributions

KS, KW and TS contributed to the study conception and design. SFS, HY, and MM participated in data collection, data analysis and interpretation, and drafted the manuscript. YI participated in data collection, data interpretation, and edited the manuscript. NM participated in data interpretation and edited the manuscript. HN and KO participated study design, data collection, data interpretation, and edited the manuscript. All authors read and approved the final manuscript.
